# Effect of Gate Bias Stress on the Electrical Characteristics of Ferroelectric Oxide Thin-Film Transistors with Poly(Vinylidenefluoride-Trifluoroethylene)

**DOI:** 10.3390/ma16062285

**Published:** 2023-03-12

**Authors:** Bon-Seong Gu, Eun-Seo Park, Jin-Hyuk Kwon, Min-Hoi Kim

**Affiliations:** 1 Department of Creative Convergence Engineering, Hanbat National University, Daejeon 34158, Republic of Korea; 2 Research Institute of Printed Electronics & 3D Printing, Industry University Cooperation Foundation, Hanbat National University, Daejeon 34158, Republic of Korea

**Keywords:** ferroelectric, oxide TFT, P(VDF-TrFE), gate bias stress, threshold voltage shift

## Abstract

We investigated the effect of gate bias stress (GBS) on the electrical characteristics of ferroelectric oxide thin-film transistors (FeOxTFTs) with poly(vinylidenefluoride-trifluoroethylene). Generally, conventional oxide thin-film transistors (OxTFTs) with dielectric gate insulators exhibit a small negative shift under negative gate bias stress (NBS) and a large positive shift under positive gate bias stress (PBS) in transfer characteristic curves. In contrast, the FeOxTFTs show a small positive shift and a large negative shift under NBS and PBS, respectively. It was confirmed that sufficient changes in the electrical characteristics are obtained by 10 min NBS and PBS. The changed electrical characteristics such as threshold voltage shift, memory on- and memory off-current were maintained for more than 168 h after NBS and 24 h after PBS. It is deduced that, since the dipole alignment of the ferroelectric layer is maximized during GBS, these changes in electrical properties are caused by the remnant dipole moments still being retained during the gate sweep. The memory on- and memory off-current are controlled by GBS and the best on/off current ratio at 10^7^ was obtained after NBS. By repeatedly alternating NBS and PBS, the electrical characteristics were reversibly changed. Our results provide the scientific and technological basis for the development of stability and performance optimization of FeOxTFTs.

## 1. Introduction

Oxide thin-film transistors (OxTFT) have attracted great attention due to their high field-effect mobility, high optical transparency, and solution processability [1,2,3,4]. In order to examine the electrical stability of OxTFTs, numerous studies about the characteristic variations caused by long-term gate bias stress (GBS), such as negative gate bias stress (NBS), positive gate bias stress (PBS), and negative bias illumination stress (NBIS), have been conducted [5,6]. In conventional OxTFTs, NBS and PBS typically induce negative and positive threshold voltage (*V*_T_) shifts, respectively [5].

Recently, various attempts have been made to exploit OxTFTs as a ferroelectric or charge trap memory device [7,8]. Ferroelectric oxide thin-film transistors (FeOxTFTs), which use ferroelectrics as a gate insulator, facilitate programing and erasing operations at relatively low voltages compared to charge trap transistors [9]. Representatives of ferroelectrics for gate insulators include lead zirconate titanate, silicon-doped hafnia, and poly(vinylidenefluoride-trifluorethylene) (P(VDF-TrFE)). Since P(VDF-TrFE) thin films can be fabricated via a facile low-cost solution process, P(VDF-TrFE) has been actively studied thus far [10,11,12]. Previously, studies on the improvement of the retention characteristics of the FeOxTFT memories with solution-processed P(VDF-TrFE) insulator have also being conducted using the solution-processed indium-gallium-zinc oxide (IGZO) semiconductor [13].

In an array architecture where multiple memory devices are integrated, a voltage for the operation of a certain cell is simultaneously applied to the gates of other constituent cells [14]. Especially, in the case of NAND-type ferroelectric memories explored recently, positive or negative read voltages should be continuously applied to the devices for the pass states of FeOxTFTs [15]. Although there is a growing need for investigating the effect of GBS in ferroelectric memory devices, GBS-induced variations in the memory characteristics of FeOxTFTs have not yet been studied.

In this work, we investigated the effect of GBS on the electrical characteristics of solution-processed FeOxTFTs with P(VDF-TrFE). First, we compared the NBS and PBS characteristics of OxTFTs in both bottom-gate and top-gate configurations applying dielectric and ferroelectric polymers. Moreover, the effect of GBS time on the electrical characteristics of the devices and the electrical sustainability of the GBS-induced characteristic changes were examined. It was found that the memory on- and off-currents can be controlled through the application of GBS, and the FeOxTFT with P(VDF-TrFE) exhibited the highest on/off current ratio at ≈10^7^ after being subjected to NBS. Lastly, the reversibility of the characteristic changes by repeatedly alternated NBS and PBS were tested.

## 2. Materials and Methods

An IGZO solution was prepared by dissolving indium nitrate hydrate, gallium nitrate hydrate, and zinc nitrate hydrate into 2-methoxyethanol with an In:Ga:Zn molar ratio of 4:3:2. The solution was stirred for 8 h at 60 °C and then filtered through a syringe filter. A poly(methyl methacrylate) (PMMA) solution was prepared by dissolving PMMA in toluene at 5 wt.%. A P(VDF-TrFE) solution was prepared by dissolving P(VDF-TrFE) (75/25) into cyclopentanone at 10 wt.%.

**Figure 1:**
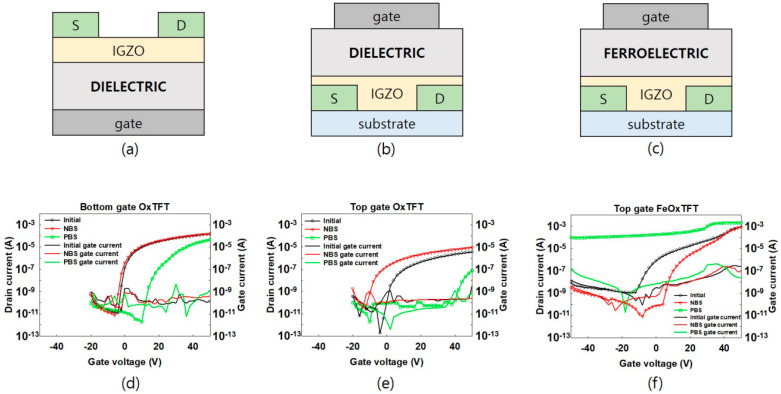
The device structures for (**a**) the conventional bottom-gate OxTFT with a dielectric gate insulator, (**b**) top-gate OxTFT with a dielectric gate insulator, and (**c**) top-gate OxTFT with a ferroelectric gate insulator. The transfer characteristic curves of (**d**) the conventional bottom-gate OxTFT with the dielectric gate insulator (i.e., silicon dioxide), (**e**) top-gate OxTFT with the dielectric gate insulator (i.e., PMMA), and (**f**) top-gate OxTFT with the ferroelectric gate insulator (i.e., P(VDF-TrFE)) after 1-h NBS and 1-h PBS.

Figure 1a–c show the schematic diagrams of the bottom-gate top-contact OxTFT with dielectric gate insulator, top-gate bottom-contact OxTFT with dielectric gate insulator, and top-gate bottom-contact FeOxTFT with ferroelectric gate insulator, respectively. For bottom-gate top-contact OxTFT (Figure 1a), the IGZO solution was first spin-coated on a substrate (i.e., heavily *p*-doped silicon with thermally grown 200 nm-thick silicon dioxide) at 3000 rpm for 30 s with subsequent two-step thermal treatment: for 10 min at 110 °C and then for 3 h at 500 °C. Lastly, 120 nm thick aluminum source/drain electrodes were thermally deposited on the IGZO layer through a shadow mask. For top-gate bottom-contact OxTFT with dielectric gate insulator (Figure 1b), the IGZO solution was first spin-coated on an indium-tin-oxide source/drain-patterned substrate at 3000 rpm for 30 s with subsequent two-step thermal treatment: for 10 min at 110 °C and then for 3 h at 500 °C. The PMMA solution was then spin-coated on the IGZO layer at 3000 rpm for 30 s with subsequent thermal treatment for 1 h at 90 °C. Lastly, a 120 nm thick aluminum gate electrode was thermally deposited on the PMMA layer through a shadow mask. For top-gate bottom-contact FeOxTFT with ferroelectric gate insulator (Figure 1c), the IGZO solution was first spin-coated on an indium-tin-oxide source/drain-patterned substrate at 3000 rpm for 30 s with subsequent two-step thermal treatment: for 10 min at 110 °C and then for 3 h at 500 °C. The P(VDF-TrFE) solution was spin-coated on the IGZO layer at 3000 rpm for 30 s with subsequent thermal treatment for 2 h at 140 °C. Lastly, a 120 nm thick aluminum gate electrode was thermally deposited on the P(VDF-TrFE) layer through a shadow mask. In the case of the devices with the top-gate configuration, since aluminum source/drain electrodes can be oxidized due to the high temperature for the IGZO thermal treatment, the indium-tin-oxide source/drain electrodes with relatively good thermal stability were used instead of aluminum source/drain electrodes.

All electrical characterizations for the transistors were carried out using a semiconductor analyzer (Hewlett Packard, HP 4551A). Gate voltages (*V*_G_) of −50 V and 50 V were applied to the transistors for NBS and PBS, respectively (Figure S1d, Supplementary Materials).

## 3. Results and Discussion

First, we compared our FeOxTFT with other OxTFTs having conventional dielectric gate insulators. Figure 1d shows the transfer characteristic curves of the bottom-gate OxTFT with conventional dielectric gate insulator after NBS and PBS. The *V*_G_ of −50 V and 50 V were applied to the devices for the NBS and PBS, respectively, for 1 h. Each transfer characteristic curve after GBS was measured by sweeping *V*_G_ from −50 to 50 V in 2 V increments at constant drain voltage (*V*_D_) = 5 V. IGZO-based TFTs usually exhibit n-type operation [16,17,18]. Our devices also clearly exhibited n-type operation. While the curve moves to the negative direction after NBS, a large positive shift (>10 V) is shown after PBS. With the PMMA gate insulator (i.e., a conventional polymer dielectric), the transfer curve of the top-gate OxTFT shows small negative and large positive shift under NBS and PBS, respectively, as shown in Figure 1e. This is because holes are trapped by NBS and electrons are trapped by PBS in field-effect transistors [19,20]. For NBS, the trapped holes that act as positive charges further accumulate free electrons in the channel region, which shifts the *V*_T_ and transfer curve of an n-type device in the negative direction. For PBS, the trapping of electrons reduces free electrons, which shifts the *V*_T_ and transfer curve in the positive direction. Moreover, the trapped electrons hinder the charge accumulation for channel formation, which further shifts the *V*_T_ and transfer curve in the positive direction (Figure S2, Supplementary Materials). Under NBS, a small electric field is applied to the gate insulator due to depletion of IGZO during NBS, resulting in only a small or negligible negative shift [21,22]. Therefore, with dielectric gate insulators, both bottom- and top-gate OxTFT show conventional transfer curve shift by GBS.

In contrast, with a ferroelectric P(VDF-TrFE) gate insulator, the device shows nonconventional transfer curve shift as shown in Figure 1f. First, after NBS, a small positive curve shift and reduced off-current were observed. For PBS, a large negative curve shift appeared, resulting in the off-current being significantly increased compared to the initial curve. That is, the FeOxTFT shows a GBS-induced characteristic change, which is opposite to the case with dielectric gate insulators. While the off-current is significantly increased by the PBS, the gate current maintained a comparable level without showing a discernible variation (Figure 1f). In addition, the linear mobility values were calculated (Figure S1, Supplementary Materials) [23].

**Figure 2:**
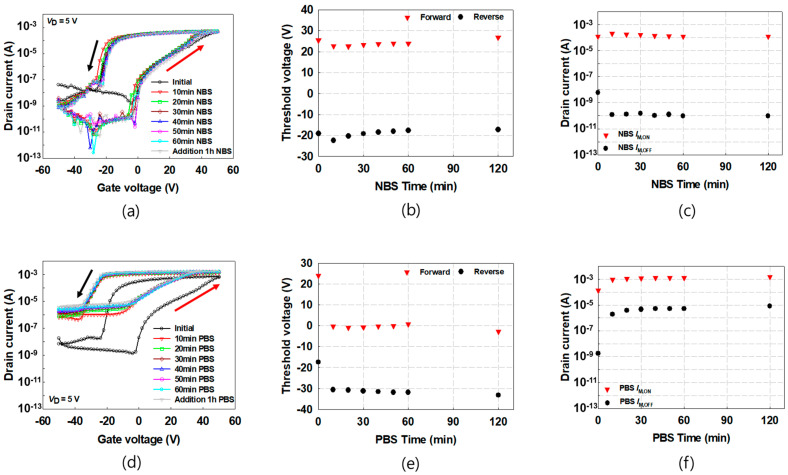
(**a**) Transfer characteristic curves, (**b**) *V*_T_, and (**c**) *I*_M,ON_ and *I*_M,OFF_ under NBS as increasing GBS time, (**d**) transfer characteristic curves, (**e**) *V*_T_, and (**f**) *I*_M,ON_ and *I*_M,OFF_ under PBS. *I*_M,ON_ and *I*_M,OFF_ are the current at *V*_G_ of −10 V during forward and backward sweeps.

In order to investigate the variation in the electrical characteristics of the FeOxTFTs according to the GBS time, the transfer characteristic curves were measured as the GBS time increases, shown in Figure 2a,d. The measurements were conducted by acquiring the transfer characteristic curves with the accumulated GBS time of 10 min, 20 min, 30 min, 40 min, 50 min, 60 min, and 120 min, under the GBS of *V*_G_ = −50 V and 50 V for the NBS and PBS, respectively. Each transfer characteristic curve after GBS was measured by sweeping *V*_G_ from −50 to 50 V and back in 2 V increments and 2 V decrements at constant *V*_D_ = 5 V. The double sweeping measurement was conducted to understand the memory characteristics of the ferroelectric transistors. As shown in Figure 2b,c,e,f, *V*_T_ at forward sweep (*V*_T,F_), *V*_T_ at reverse sweep (*V*_T,R_), memory on-current (*I*_M,ON_), and memory off-current (*I*_M,OFF_) of the transfer curves under NBS and PBS were obtained, respectively. Here, we defined *V*_T,F_ and *V*_T,R_ as a voltage where the drain current is 15 μA during forward and backward sweeps [24]. *I*_M,ON_ and *I*_M,OFF_ were defined as the current values where the *V*_G_ is −10 V during forward and backward sweeps. Under NBS, *V*_T,F_ and *V*_T,R_ shows only small changes as seen in Figure 2b, and *I*_M,ON_ exhibits negligible change shown in Figure 2c. However, *I*_M,OFF_ shows a large decrease even under 10 min of NBS and saturated with additional GBS thereafter. In contrast, when PBS is applied, *V*_T,F_ and *V*_T,R_ were moved to the negative direction more than about 15 V as shown in Figure 2e. In Figure 2f, *I*_M,ON_ and *I*_M,OFF_ are increased, and notably a large increase of about 10^3^ times was observed. That is, when NBS and PBS are applied, unlike the OxTFT with dielectric gate insulator showing negative and positive shift, the FeOxTFT rather shows positive and negative shift respectively. Note again that, in the OxTFT with dielectric gate insulators, the GBS-induced characteristic change originated from the trapped charges by the gate field. However, it has been reported that trap density in P(VDF-TrFE) interfaced with the oxide semiconductor was low [25]. In addition, due to the effect of the remnant ferroelectric dipole moments, counterclockwise hysteresis, rather than clockwise hysteresis, appears by trapped charges. During the *V*_G_ sweeping in the transfer characteristic measurement, which proceeds for a short time (<15 s), ferroelectric dipole moment alignment is not achieved in the entire ferroelectric layer. During *V*_G_ sweeping, the ferroelectric dipole moments are aligned according to the gate electric field in the relatively high crystalline region. However, under long GBS, the additional ferroelectric dipole moments are aligned even in the region where the alignment of ferroelectric dipole moments is difficult under short gate bias. The GBS possibly suppresses partial disorder in the P(VDF-TrFE) layer, which leads to the alignment of the additional ferroelectric dipole moments [26]. After this GBS, if a *V*_G_ is swept for a short time, the additional ferroelectric dipole moments aligned by the previous GBS do not change along the *V*_G_ (Figure S3, Supplementary Materials). For example, under PBS, ferroelectric dipole moments are formed in an upward direction in our top-gate FeOxTFTs. During the gate sweep after PBS, even if the *V*_G_ becomes negative, exceeding the coercive voltage, the additional ferroelectric dipole moments maintain an upward direction instead of a downward direction (Figure S2, Supplementary Materials). Therefore, by these unswept ferroelectric dipole moments, the drain current is increased and the transfer curve is negatively shifted.

**Figure 3:**
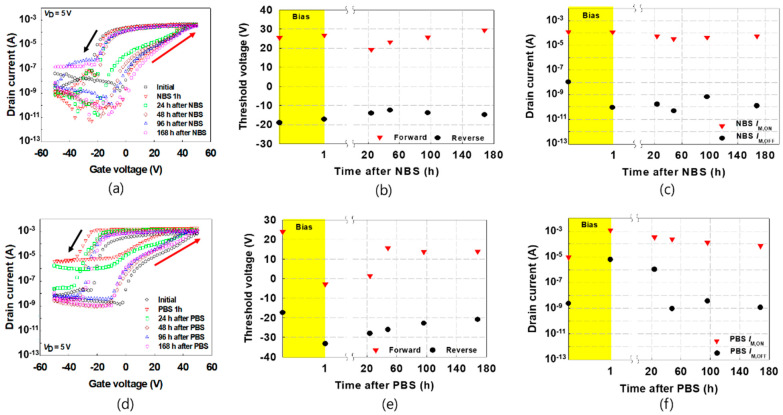
(**a**) Transfer characteristic curves, (**b**) *V*_T_, (**c**) *I*_M,ON_ and *I*_M,OFF_ according to elapsed time after NBS, (**d**) transfer characteristic curves, (**e**) *V*_T_, (**f**) *I*_M,ON_ and *I*_M,OFF_ according to elapsed time after PBS.

In order to find out the electrical sustainability of the GBS-induced characteristic changes (i.e., how long the changed properties of the FeOxTFTs due to GBS are maintained), the transfer characteristic curves were measured corresponding to the elapsed time as shown in Figure 3a,d. After applying the GBS of *V*_G_ = −50 V and 50 V for the NBS and PBS, respectively to the devices, the transfer characteristic curves were acquired with the elapsed time of 1 h, 24 h, 48 h, 96 h, and 168 h. The transfer characteristics were measured by sweeping *V*_G_ from −50 to 50 V and back in 2 V increments and 2 V decrements at constant *V*_D_ = 5 V. For the case of NBS, since the change of *V*_T,F_, *V*_T,R_, and *I*_M,ON_ by NBS were small, a significant change according to the elapsed time was not observed; see Figure 3b,c. The value of *I*_OFF_ significantly reduced by NBS was maintained for 168 h. For the case of PBS, *V*_T,F_, *V*_T,R_, *I*_M,ON_, and *I*_M,OFF_, which showed large changes due to PBS, varied close to the initial values as time passed, as shown in Figure 3e,f. In detail, after 24 h, the characteristics of FeOxTFT changed by PBS are maintained to some extent, but after 96 h, the values become similar to the transfer curve values in the initial state.

**Figure 4:**
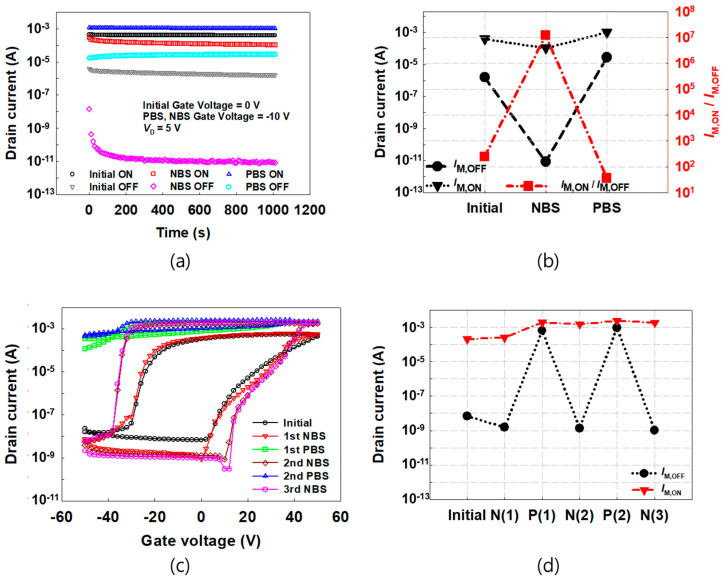
(**a**) Retention characteristics of the FeOxTFT initial and after NBS and PBS. (**b**) *I*_M,ON_, *I*_M,OFF_, and *I*_M,ON_/*I*_M,OFF_ of the FeOxTFT initial after 1000 s. (**c**) Transfer characteristic curves. (**d**) The *I*_M,ON_ and *I*_M,OFF_ of the initial FeOxTFT and those subjected to repeatedly alternated NBS and PBS.

To investigate the memory characteristics of the FeOxTFTs, the retention characteristics of *I*_D_ were measured as shown in Figure 4a. The device was programmed by applying the high *V*_G_ of 50 V and −50 V, and then, the *I*_M,ON_ and *I*_M,OFF_ were measured with the *V*_G_ of 0 V for the initial measurement and with the *V*_G_ of −10 V for the post-NBS and post-PBS measurements. The *V*_G_ of −50 V and 50 V for the NBS and PBS, respectively, were applied to the device for 1 h. *I*_M,ON_ and *I*_M,OFF_ were well retained during 1000 s for the initial (no bias), NBS, and PBS cases. It was noted that in the case of NBS, *I*_M,OFF_ shows a decreasing value over time, which occurs when the holes charged in ferroelectric in erasing operation are discharged during retention measurement. *I*_M,ON_, *I*_M,OFF_, and *I*_M,ON_/*I*_M,OFF_ of the FeOxTFTs after 10^3^ s are presented in Figure 4b. In the initial FeOxTFT, *I*_M,ON_ and *I*_M,OFF_ shows 388.9 μA and 1.6 μA, respectively, and thus an *I*_M,ON_/*I*_M,OFF_ value of 2.5 × 10^2^ is obtained. In the case of NBS, a significantly improved *I*_M,ON_/*I*_M,OFF_ value of 10^7^ is shown due to largely reduced *I*_M,OFF_. For PBS, while *I*_M,ON_ increases, *I*_M,OFF_ increases excessively, resulting in a reduced *I*_M,ON_/*I*_M,OFF_ value of 0.4 × 10^2^ compared to the NBS case.

In order to study the reversibility of the GBS effect, the NBS and PBS were applied alternately and repeatedly as shown in Figure 4c. The transfer characteristic curves were obtained with applying the first NBS (GBS #1), first PBS (GBS #2), second NBS (GBS #3), second PBS (GBS #4), and third NBS (GBS #5) in sequence. In Figure 4d, *I*_M,ON_ and *I*_M,OFF_ repeatedly decrease after NBS and increase after PBS. This means that the direction of the unswept ferroelectric dipole moments during the gate sweeping can be controlled reversibly by GBS (Figure S3, Supplementary Materials). The repeatedly alternated NBS and PBS further suppressed the partial disorder [26], which eventually enabled the reversible rotation of the additional ferroelectric dipole moments. In a real memory array circuit, a larger value of *I*_M,ON_ and *I*_M,OFF_ or a larger value of *I*_M,ON_/*I*_M,OFF_ is preferred according to the number of memory elements connected together and the connected circuit in the next stage. Therefore, using NBS and PBS, the performance of the FeOxTFTs is reversibly optimized. Future work needs to investigate the origin of the partial disorder in P(VDF-TrFE) layers and the mechanism for the alignment of additional ferroelectric dipole moments.

## 4. Conclusions

The FeOxTFT with a P(VDF-TrFE) gate insulator exhibited small variations in *V*_T_ and *I*_M,ON_, and a large reduction in *I*_M,OFF_ under the NBS, while exhibiting a large negative shift in *V*_T_, and large increases in *I*_M,ON_ and *I*_M,OFF_ under the PBS. The large variations in *V*_T_, *I*_M,ON_, and *I*_M,OFF_ under the GBS were attributed to the alignment of the additional ferroelectric dipole moments in the P(VDF-TrFE) layer. In the sustainability tests, the *V*_T_, *I*_M,ON_, and *I*_M,OFF_ changed by the NBS were maintained for more than 168 h, and those changed by the PBS for 24 h. In the retention tests, the *I*_M,ON_, and *I*_M,OFF_ of the initial FeOxTFT and those subjected to the NBS and PBS were well retained for 1000 s. Moreover, the *I*_M,ON_ and *I*_M,OFF_ of the FeOxTFT tended to increase and to decrease, respectively, with the application of the repeatedly alternated NBS and PBS. This indicates that the reversibility of the GBS effect was obtained by repeatedly alternating the NBS and PBS. These results will contribute to improving the electrical stability and performance of FeOxTFTs.

## Data Availability

The data presented in this study are available on request from the corresponding author. The data are not publicly available due to privacy.

## Supplementary Materials

The following supporting information can be downloaded at: https://www.mdpi.com/article/10.3390/ma16062285/s1, Figure S1: (a–c) The device structures, (d) the circuit diagram for the GBS, and the linear-scale transfer characteristic curves of (e) the conventional bottom-gate OxTFT with the dielectric gate insulator (i.e., silicon dioxide), (f) top-gate OxTFT with the dielectric gate insulator (i.e., PMMA), and (g) top-gate OxTFT with the ferroelectric gate insulator (i.e., P(VDF-TrFE)) after 1-h NBS and 1-h PBS; Figure S2: The energy band diagrams for the OxTFT and FeOxTFT subjected to the PBS and NBS; Figure S3: Schematic diagrams for the ferroelectric dipole changes in the P(VDF-TrFE) layer in the PBS and NBS cases.
