# Supplementary material for: Effect of Gate Bias Stress on the Electrical Characteristics of Ferroelectric Oxide Thin-Film Transistors with Poly(Vinylidenefluoride-Trifluoroethylene)
Source: Materials (Basel). 2023 Mar 12;16(6):2285. doi: 10.3390/ma16062285 (PMC10057146; doi:10.3390/ma16062285)
Supplement: Supplementary file 1 [file materials-16-02285-s001.zip › materials-2233701-supplementary.pdf]

Supplementary Materials

Effect of Gate Bias Stress on the Electrical Characteristics of Ferroelectric Oxide Thin-Film Transistors with Poly(Vinylidenefluoride-Trifluoroethylene)

Bon-Seong Gu[1], Eun-Seo Park[1], Jin-Hyuk Kwon[2,*] and Min-Hoi Kim[1,2,*]

[1]*Department of Creative Convergence Engineering, Hanbat National University, Daejeon 34158, Republic of Korea*

[2] *Research Institute of Printed Electronics & 3D Printing, Industry University Cooperation Foundation, Hanbat National University, Daejeon 34158, Republic of Korea*

* Correspondence: jhkwon@hanbat.ac.kr (J.-H.K.); mhkim8@hanbat.ac.kr (M.-H.K.); Tel.: +82-42-821-1973 (M.-H.K.)

Figure S1d shows the circuit diagram for the GBS (i.e., NBS and PBS) before measuring each transfer characteristic curve. Figure S1e−g show the transfer characteristic the conventional bottom-gate OxTFT with the dielectric gate insulator (i.e., silicon dioxide), top-gate OxTFT with the dielectric gate insulator (i.e., PMMA), and top-gate OxTFT with the ferroelectric gate insulator (i.e., P(VDF-TrFE)) after 1-h NBS and 1-h PBS, respectively in linear scale. The linear mobility (μ_{lin}) was extracted from the slope of the drain current (I_D) *vs*. gate voltage (V_G) plots, based on the following equation: $\mu_{\mathrm{lin}} = \frac{dI_{\mathrm{D}}}{dV_{\mathrm{G}}}\left(\frac{LC_{\mathrm{i}}}{WV_{\mathrm{D}}}\right)$ where C_i is the gate-insulator capacitance and V_D the drain voltage. For the conventional bottom-gate OxTFT with the dielectric gate insulator (i.e., silicon dioxide), the μ_{lin} values were 31.5 cm^2/Vs (initial), 32.8 cm^2/Vs (after NBS), and 20.2 cm^2/Vs (after PBS), respectively. For the top-gate OxTFT with the dielectric gate insulator (i.e., PMMA), the μ_{lin} values were 0.8 cm^2/Vs (initial), 1.5 cm^2/Vs (after NBS), and 0.1 cm^2/Vs (after PBS), respectively. For the top-gate OxTFT with the ferroelectric gate insulator (i.e., P(VDF-TrFE)), the μ_{lin} values are presented in Figure S1g. These μ_{lin} values are considered unreasonable, since they were possibly over- or under-estimated due to dipole changes in the P(VDF-TrFE) layer during the V_G sweep.

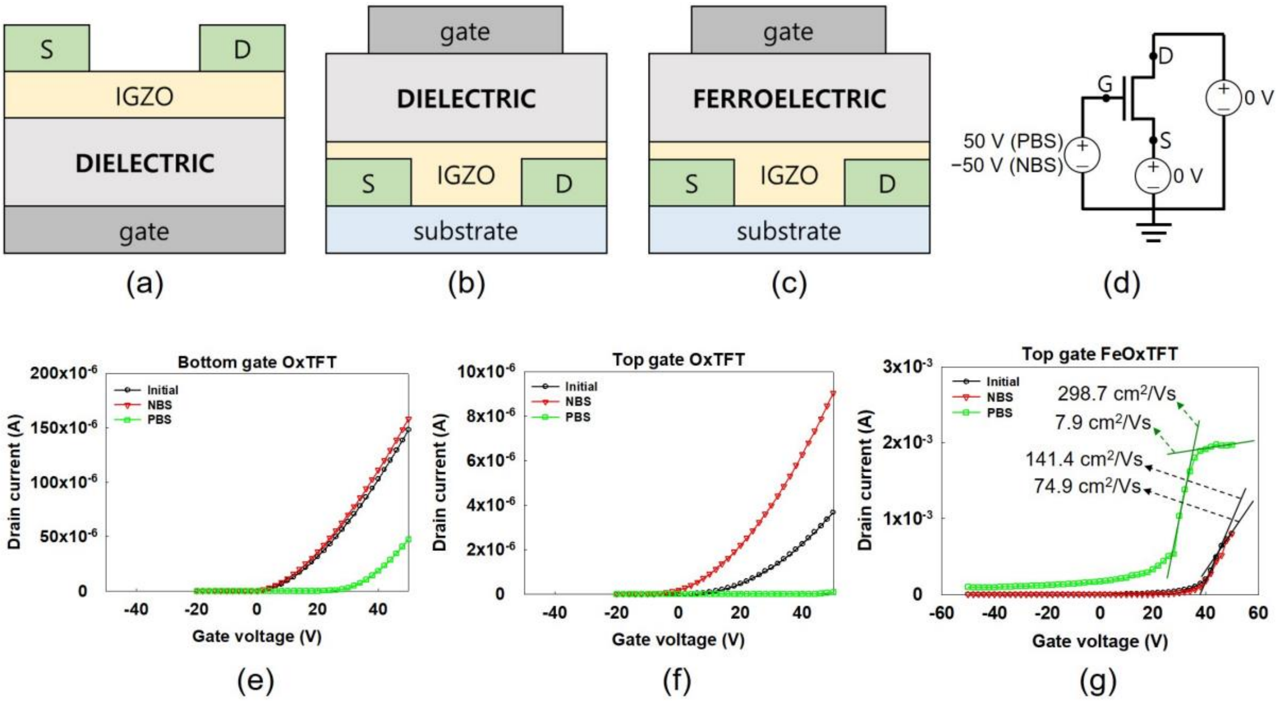

Figure S1. The device structures for (a) the conventional bottom-gate OxTFT with a dielectric gate insulator, (b) top-gate OxTFT with a dielectric gate insulator, and (c) top-gate OxTFT with a ferroelectric gate insulator. (d) The circuit diagram for the GBS. The transfer characteristic curves of (e) the conventional bottom-gate OxTFT with the dielectric gate insulator (i.e., silicon dioxide), (f) top-gate OxTFT with the dielectric gate insulator (i.e., PMMA), and (g) top-gate OxTFT with the ferroelectric gate insulator (i.e., P(VDF-TrFE)) after 1-h NBS and 1-h PBS.

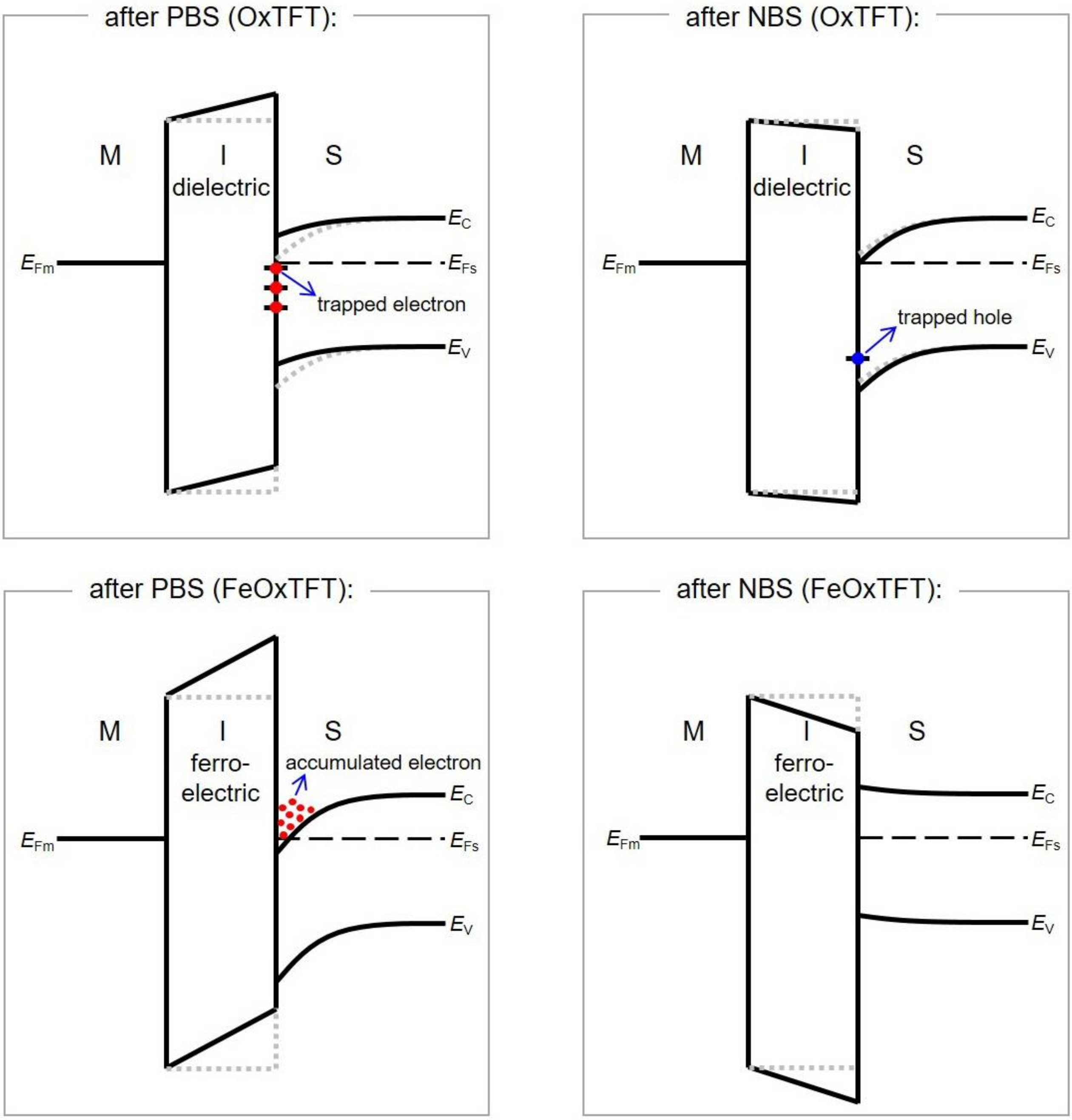

Figure S2. The energy band diagrams for the OxTFT and FeOxTFT subjected to the PBS and NBS.

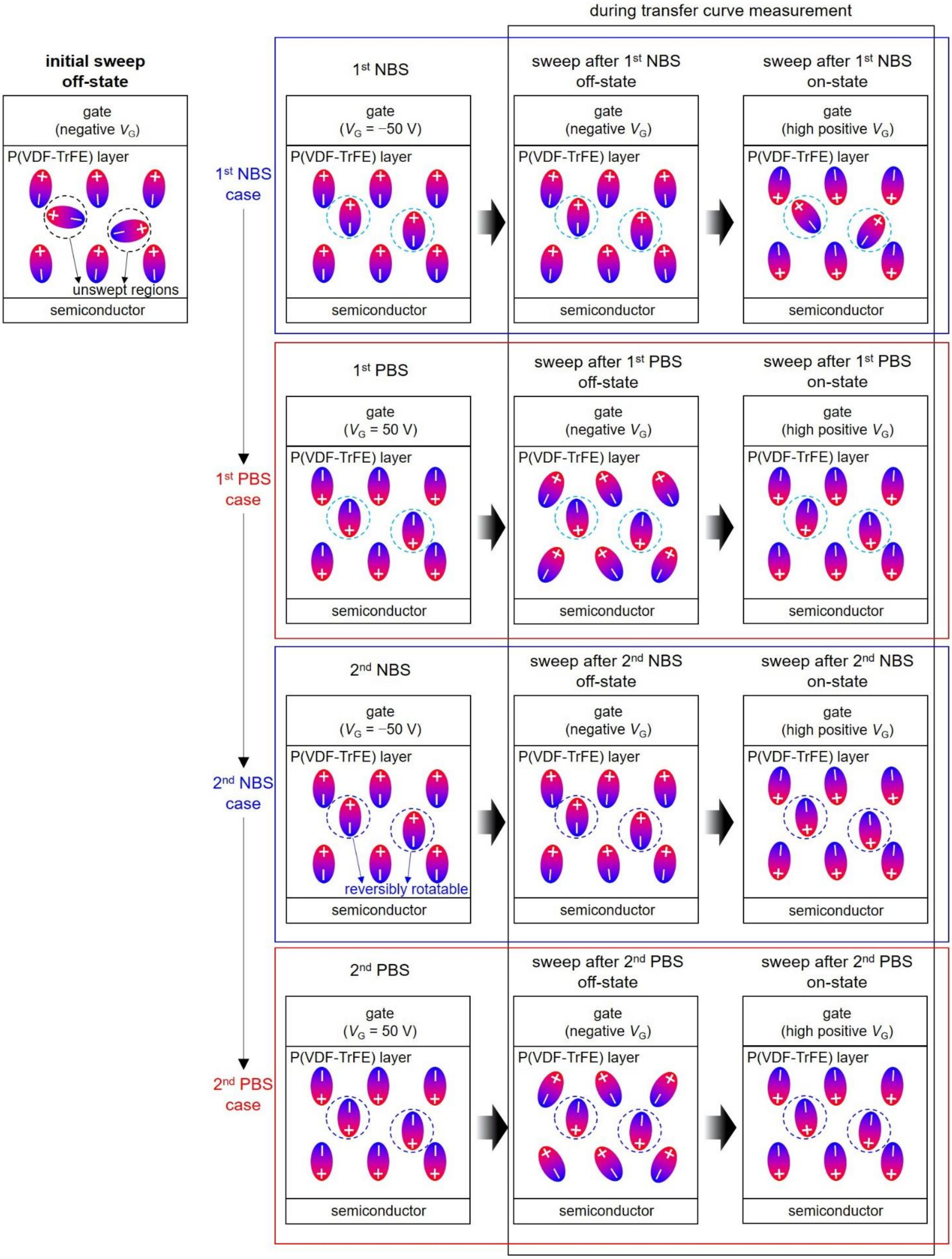

Figure S3. Schematic diagrams for the ferroelectric dipole changes in the P(VDF-TrFE) layer in the PBS and

NBS cases.
